# Brain temperature as proxy for brain state and oscillatory activity in the mouse

**DOI:** 10.1038/s41598-025-21175-3

**Published:** 2025-10-24

**Authors:** Andrey Lazopulo, Yann Emmenegger, Nina Đukanović, Marieke M.B. Hoekstra, Paul Franken

**Affiliations:** 1 Center for Integrative Genomics, University of Lausanne, Lausanne, Switzerland; 2 VIB Center for Brain & Disease Research, KU Leuven, Leuven, Belgium

**Keywords:** Neuroscience, Physiology

## Abstract

Brain temperature and brain activity are in a complex, bidirectional relationship. Changes in brain temperature impact brain functioning and, conversely, brain activity generates heat. The latter can be illustrated by the characteristic changes in brain temperature that accompany the transitions between the brain states wakefulness, NREM sleep, and REM sleep. Here we show in the mouse that these typical temperature changes are sufficiently consistent to predict brain state. To gain further insight into this relationship, we quantified the effects of specific EEG activity patterns characteristic of sleep-wake states on temperature. We found that occurrences of spindles (11–15 Hz) during NREM sleep and of theta (7–9 Hz) and gamma (55–85 Hz) activity during wakefulness and REM sleep, were followed by increases in cortical temperature with a 10–14 s delay. In contrast, temperature decreased during the theta-rich cataplexy-associated state (CAS) observed in mice lacking the *hypocretin* gene, shedding new light on this non-physiological state. Our results show that brain temperature can be used as a reliable and accessible proxy of brain state and the accompanying oscillatory activity.

## Introduction

The profound effects of temperature on biochemical and physiological processes have been well documented^1^. In brain tissue, temperature influences a wide range of neuronal functions including nerve conduction, membrane potential, synaptic transmission, and spiking activity^2–4^. Several studies assessed the effects of brain temperature on sleep-related neural dynamics. For example, warming cortical samples reduced the duration of the neuronal upstate and increased firing rates during upstates, while cooling produced the opposite effects^5^. Others demonstrated that heating the mouse thalamocortical circuitry in vivo increases spindle frequency^6^. Overall brain activity, as assessed by the EEG, also depends on changes in brain temperature such that decreases in temperature slow down the period of rhythmic brain activity and reduce EEG amplitude^7^. While the changes in temperature on brain activity are widely recognized, how EEG-defined brain states and specific activities characteristic of sleep-wake states affect brain temperature is less well studied.

Neuronal processes are metabolically demanding^8^. In humans approximately 20% of total oxygen consumption is used by the brain, although it represents only 2% of our total body mass^9^. Similarly, cerebral metabolic rate in the rat was found to be more than 6-times larger than that in other metabolically active tissues such as skeletal muscle^10^. During neuronal activity, some of the energy consumed is converted into heat and multiple studies have demonstrated that neurons generate heat when firing action potentials^11^. Also, at the level of the whole brain, neuronal activity increases temperature. For example, the introduction of novel objects or tail pinch produced an increase in brain temperature in rats^12^ and short visual or auditory stimulation increased brain temperature in cats^13^. Differences in neuronal activity are likely to be a major contributor to the observed brain temperature difference between sleep-wake states.

Prior research has shown that changes in brain temperature are tightly coupled to changes in sleep-wake states in a variety of bird and mammalian species^14–20^. Moreover, we have shown in rats and mice that both the short-term changes in brain temperature at sleep-wake transitions as well as its daily fluctuations can be predicted with high accuracy based on the sequence of sleep-wake states with little direct influence of circadian factors^19,20^. To further explore the tight relationship between brain state and brain temperature, we investigated whether variations in brain temperature are sufficiently consistent to predict the occurrence of waking, NREM sleep, and REM sleep in mice. In addition to these three physiological brain states, we assessed temperature dynamics during cataplexy, the most striking symptom of the wake disorder type-1 narcolepsy that we have shown in the mouse to be composed of a predictable sequence of EEG activities, including a distinct cataplexy-associated state^21^. To extend these findings to specific brain activities, we analyzed the temperature changes at discrete EEG events characteristic of each sleep-wake state. We found that EEG events known to be associated with high neuronal activity resulted in temperature increases. Our findings demonstrate that temperature changes can be used as a reliable and easy to measure proxy of neuronal activity that can be recorded at high temporal resolution.

## Results

### Changes in cortical temperature predict sleep-wake states

**Fig. 1:**
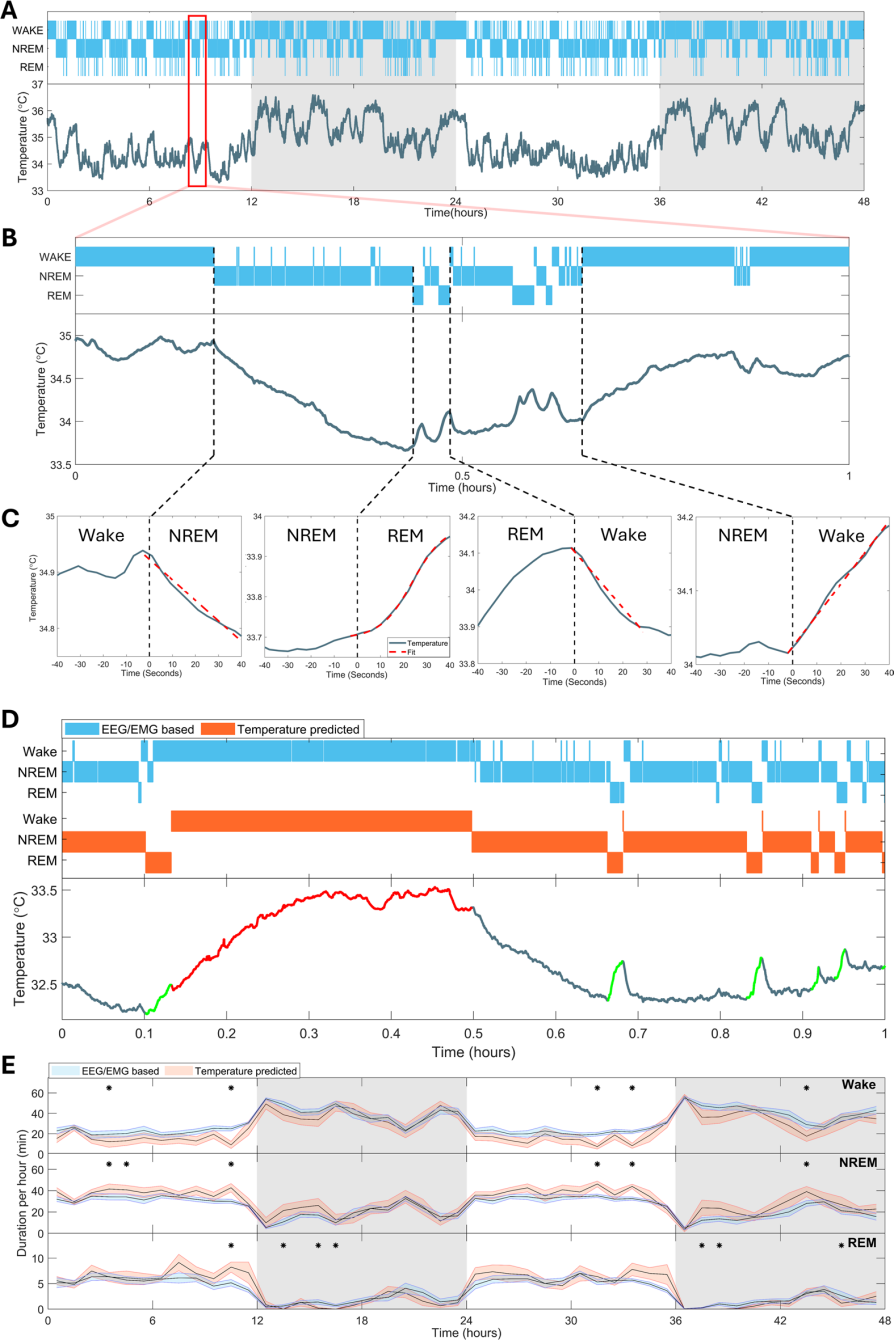
Changes in cortical temperature predict sleep-wake state transitions. (**A**) Example of a 48-hour recording of sleep-wake states (top) and cortical temperature (bottom) in a mouse. (**B**) Zoom in of the recording illustrates the typical changes in temperature at sleep-wake state transitions. (**C**) Example of temperature changes at transitions between Wake, REM sleep, and NREM sleep (blue lines). The decreases in temperature during NREM sleep following Wake (left) and during Wake following REM sleep (2nd right), and its increase during Wake following NREM sleep (right panel) initially follow approximately linear functions (red dashed lines). The temperature changes during REM sleep after NREM sleep followed a sigmoidal dynamic (2nd left panel). (**D**) Example of algorithm performance. Parts of a temperature recording used to detect REM sleep and Wake are highlighted (red – Wake, green – REM sleep). The algorithm is not able to detect episodes shorter than 20 s that do not produce interpretable temperature changes. (**E**) Comparison of temperature-based predictions with EEG-EMG based sleep-wake states. The hourly values calculated from the algorithm output (blue line) and EEG-EMG defined scoring (red line) agree for 87% of the hourly intervals. Black dots above the curves mark hourly intervals in which predicted and observed time-in-state differed significantly (Mann-Whitney test, *p* < 0.05).

We quantified EEG-EMG determined sleep-wake states and cortical temperature continuously for 48 h in 15 mice. The data not only confirmed the typical nychthemeral changes in brain temperature over the two days, i.e., high in the dark or active phase and low during the light or rest phase, but also the typical changes occurring immediately after sleep-wake state transitions (Fig. 1A-C). For instance, an approximately linear decrease in temperature occurred in the ca. first 30 s after the transition from waking to NREM sleep as well as from REM sleep to wake. Transitions from NREM sleep to wakefulness or to REM sleep were both associated with a rapid increase in cortical temperature, which was linear during wakefulness but followed a sigmoidal time course in REM sleep (Fig. 1C).

To test whether these temperature dynamics can predict sleep-wake state transitions, we developed an algorithm that searches for the abovementioned characteristic changes in temperature (see Methods for details). First episodes of wakefulness were identified. Waking episodes ended when followed by a continuous > 5 s decrease in temperature and started with an increase that brought the temperature to the levels observed at the end of the waking episode. Then REM sleep episodes were identified. A sigmoid function was fitted to temperature data using a 2 s sliding window. REM sleep started when the temperature change within a 2 s window fitted the sigmoid (R^2^ > 0.97), and ended by a continuous > 0.4 s drop in temperature. The remainder was considered NREM sleep. Our approach relied on consistent decreases or increases in temperature (Fig. 1C) and therefore sleep-wake episodes shorter than 20 s could not be captured (Fig. 1D). Despite this drawback, the hourly values of time spent in state determined by the algorithm did not importantly deviate from the EEG-EMG derived sleep-wake data and only in ~ 12% of the 1 h intervals a significant difference was obtained (Fig. 1E). Because short wake and REM sleep episodes were labeled as NREM sleep the algorithm overestimated this state.

These results extend earlier observations that brain states are strongly associated with typical and predictive temperature dynamics. The tight coupling between the two is likely to relate to the differences in neuronal activity between sleep-wake states in the cortex^22,23^. This suggests that increases in cortical temperature primarily reflect the heat production associated with increases in average neuronal firing^24^. To address this hypothesis, we next assessed whether temperature changes followed specific cortical activities within sleep-wake states.

### High sleep spindle incidence increases cortical temperature

**Fig. 2:**
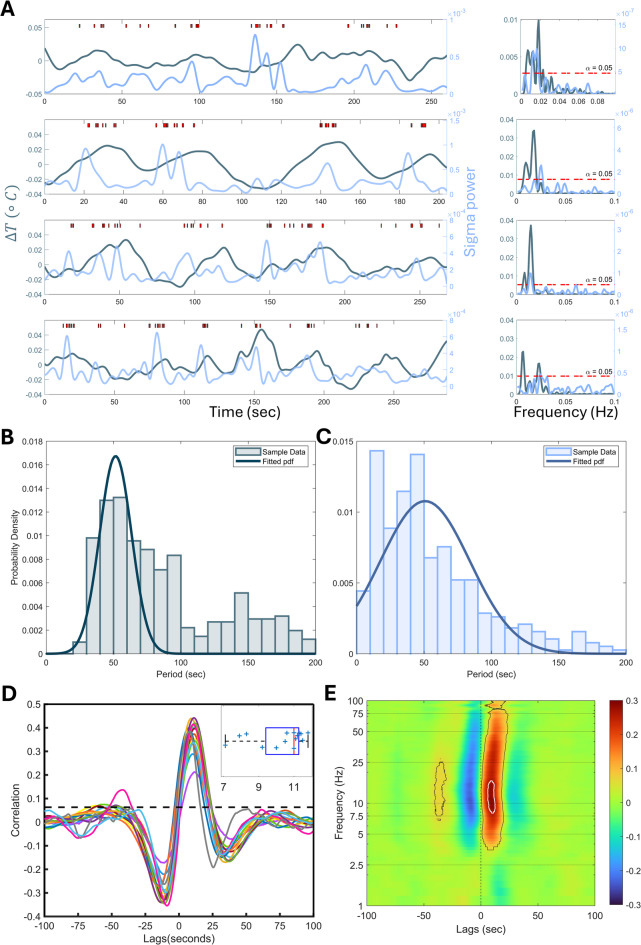
Periods of high spindle density during NREM sleep are followed by an increase in brain temperature. (**A**) The four graphs on the left each show an example of cortical temperature (dark blue) and sigma power (10–15 Hz; light blue) and the occurrence of sleep spindles (red ticks) during a NREM sleep episode. Data taken from 4 different mice at different times of the 24 h day. Right-hand panels show the EEG power spectra calculated for the signals on the left using the Lomb-Scargle periodogram. The red-dashed lines indicate the *p* = 0.05 significance levels (Lomb-Scargle function). (**B**) Histogram of period lengths extracted from temperature power spectra for NREM sleep episodes (> 200 s) in which rhythmicity was significant (*p* < 0.05; ~12 episodes/mouse). The histogram points to the presence of a ca. 50 s periodicity in temperature. (**C**) Same as B but for sigma power. The histogram of periods detected in sigma power changes showed the expected peak at ca. 50 s. (**D**) Cross-correlations between changes in temperature and in sigma power. Peaks larger than $\:2/\surd\:N$ (dashed line; where *N* is number of data points, *N* = 1000 in the shortest considered epoch), show a significant correlation. The average correlation within each individual mouse (one line/mouse; *n* = 15) was calculated from NREM sleep episodes > 100 s (~ 134 episodes/mouse). Temperature changes showed a delay that varied between 7 and 12 s (µ = 10.26 s, σ = 1.46 s; see inset) with respect to sigma power. (**E**) Cross-correlations for the entire EEG frequency range (0–100 Hz, 0.25 Hz resolution; note the non-linear scale). Correlations for each frequency band were averaged over all NREM sleep episodes from all animals shown in D and combined into one heatmap with the correlation strength given by the color. Black contour lines delimit correlations >$\:2/\surd\:N$. EEG activity over a wide range of frequencies correlated with changes in cortical temperature with the strongest region (> 0.25, shown with white contour) between 7.5 and 18.5 Hz.

We first examined whether spindle activity, one of the EEG hallmarks of NREM sleep^25^, is associated with changes in cortical temperature. Instances of high spindle density and resulting peaks in sigma (11–15 Hz) power occur every ca. 50 s^26^. This 50 s periodicity, referred to as the ‘infraslow oscillation’, was also reported in the thalamus of anesthetized and naturally sleeping mice where it was found to be associated with local changes in temperature^6^. By analyzing NREM sleep episodes longer than 200 s, allowing the detection of significant ∼50 s rhythmicity, we could confirm these observations at the level of the cerebral cortex (Fig. 2A). The distribution of the periodicities obtained within NREM sleep episodes with significant peaks in the Lomb-Scargle periodogram (see Methods), yielded a mean period close to 50 s both for the fluctuations in sigma power (µ = 50.14 s, σ = 31.07 s; Fig. 2C) and in cortical temperature (µ = 50.92 s, σ = 15.10 s; Fig. 2B).

We then asked how these changes in cortical temperature related to changes in spindle prevalence within individual NREM sleep episodes using EEG sigma power as a proxy for spindle prevalence^26^. Cross-correlations revealed that peaks in sigma power led peaks in cortical temperature by approximately 10 s (Fig. 2D). To investigate the specificity of EEG frequencies in which power density is associated with changes in cortical temperature, we computed the cross-correlation over the full EEG spectrum (0–100 Hz). While the strongest correlations (*r* > 0.25) centered around the sigma band, i.e., 7.5–18.5 Hz, significant correlations between EEG activity and temperature with a similar time lag were observed over a wide range of frequencies (Fig. 2E). These findings demonstrate that cortical temperature increases in response to high spindle density which might relate to the sleep-spindle related increase in neuronal activity of cortical neurons^27^.

### Fluctuations in cortical temperature during REM follow changes in theta and gamma activity

**Fig. 3:**
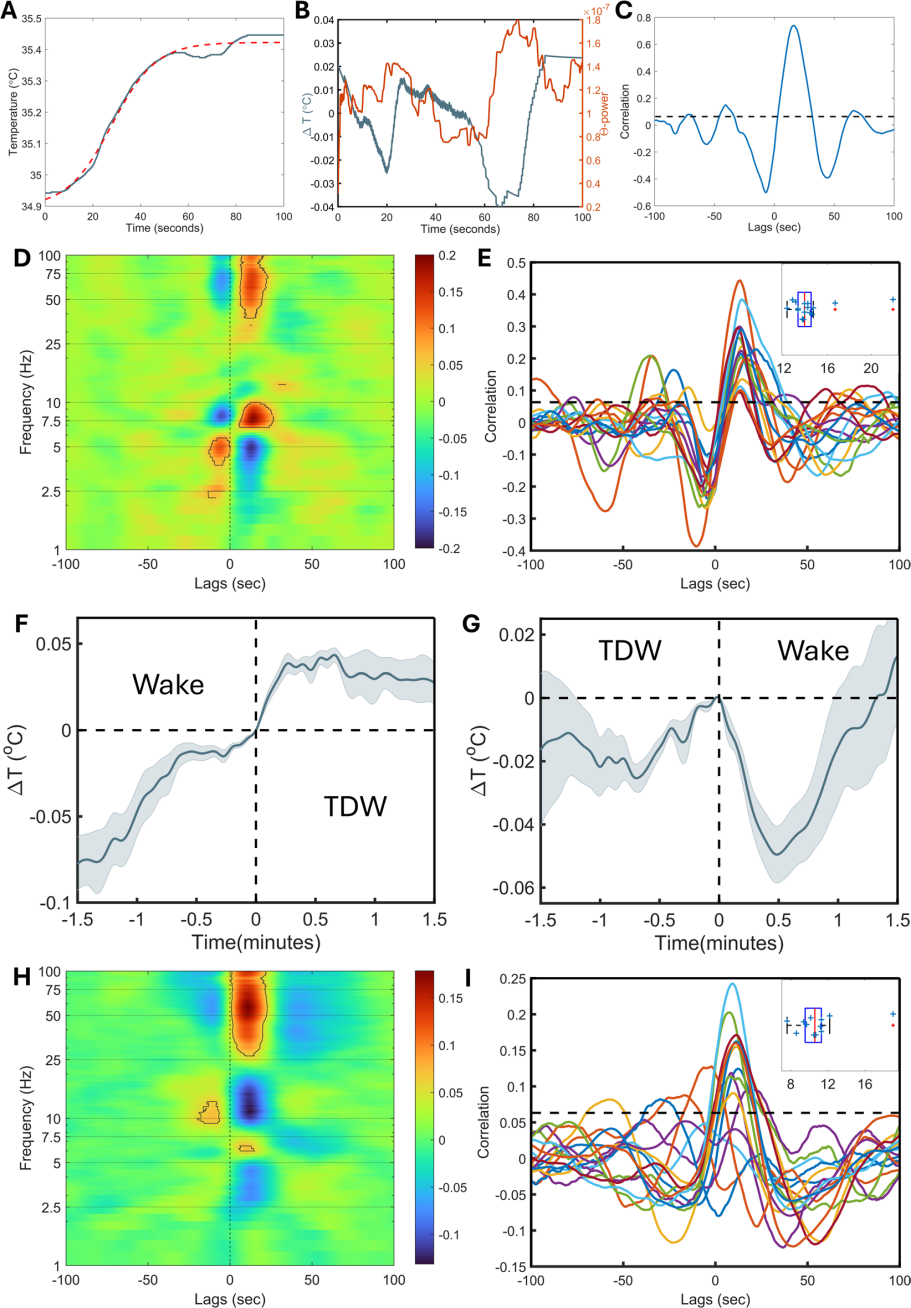
Cortical temperature changes correlate with gamma and theta in the EEG during REM sleep and TDW. (**A**) The temperature increases during REM sleep (blue line) masks subtler variations that were revealed by fitting and subtracting a sigmoid function (red dashed line). (**B**) The residual temperature changes (blue line) followed fluctuations in theta power (6–10 Hz, orange line). (**C**) The similarity and phase lag between the two signals were estimated by cross-correlation, which in the example depicted in A and B, peaked at a ca. 11 s lag between the temperature changes and theta power. (**D**) Cross-correlation between temperature and power density for the full EEG spectrum (0–100 Hz), with highest associations observed in the gamma (55–85 Hz) and theta (7.5–8.5 Hz) bands (episodes > 100 s; 20/mouse, *n* = 15). (**E**) Average cross-correlations within mice showed lags between 12 and 22 s (µ = 14.43 s, σ = 2.25 s; see inset). (**F**) Transitions from regular wakefulness to TDW were followed by an increase in cortical temperature. (**G**) Those from TDW to wakefulness were followed by a sharp decrease. (**H**) We combined all wakefulness and calculated the cross-correlation between the cortical temperature and spectral power of EEG. Similar to REM sleep, the highest association was observed for the gamma (55–85 Hz) and theta (6.5–7.5 Hz) ranges (episodes > 100 s; 80/mouse, *n* = 15). We also observed a strong negative correlation with the sigma power band, resulting from a decrease in sigma power when theta and gamma activity increase. (**I**) To estimate the delay between neuronal activity and temperature change, we focused on theta band. Peaks of correlation vary between 8 and 19 s (see inset) with an average of 11 s (µ = 10.98 s, σ = 2.85 s). Each line represents an individual mouse. In (**E**) and (**I**) peaks larger than $\:2/\surd\:N$ (dashed line; where *N* is number of data points), show a significant correlation. In (**D**) and (**H**) black contours show correlation greater than $\:2/\surd\:N$.

REM sleep is not a homogenous state and can be further subdivided into tonic and phasic substates with varying levels of neural activity that are associated with changes in theta and gamma activity^28,29^. Thus, we investigated brain temperature variations during REM sleep episodes and their relationship with varying EEG activity (Fig. 3A-E). The temperature changes during REM sleep were dominated by the large sigmoid increase (see Fig. 1C). On this rising background, we observed more subtle temperature variations, which we found to be preceded by changes in theta power (6–10 Hz), the dominant EEG activity in REM sleep in the mouse^30^, with an 11 s lag for the example REM sleep episode illustrated in Fig. 3A-C. Analyses of the full EEG frequency range for all REM sleep episodes > 100 s (*n* = 306) revealed that the temperature fluctuations correlated strongest with changes in EEG activity in the 7.5–8.5 Hz range (Fig. 3D) with a 14 s delay (µ = 14.4 s, σ = 2.25 s; Fig. 3E). Besides theta, temperature correlated also with activity in the high gamma range (55–85 Hz) with similar delay (Fig. S3A), further attesting to the tight coupling of the activity in these two frequency bands during REM sleep^31^.

### Theta-dominated waking shows higher cortical temperature

As in REM sleep, high theta activity is also the dominant EEG activity during theta-dominated wakefulness (TDW), a state linked to goal-driven and exploratory behavior^32,33^. As TDW is considered a neuronally more active state than waking without theta, we expected cortical temperature to increase. Indeed, as observed with transitions into REM sleep, brain temperature increased at transitions from wake to TDW, albeit to a lesser degree (Fig. 3F). Because temperature changes within a state depend on the temperature at which the state is initiated^34^, this smaller increase is likely to result from the higher levels reached in the state preceding TDW (i.e., wakefulness) compared to those reached in the state preceding REM sleep (i.e., NREM sleep). Temperature initially decreased after TDW ended (Fig. 3G), reminiscent of the decrease observed during the wakefulness following REM sleep (Fig. 1C, Fig. S1A).

Next, we determined whether fluctuations in temperature during TDW followed activity in specific EEG frequencies (Fig. 3H). Like in REM sleep, the highest correlation was observed in the gamma and theta frequencies. We found, however, that the theta frequencies that showed the highest correlation were in the 6.0–7.5 Hz range, i.e., left-shifted by 1 Hz compared to REM sleep (see Fig. 3D) and also slower than the dominant theta frequency in TDW (8.1 Hz). The best correlation in the gamma range was again found between 55 and 85 Hz. To determine the delay between temperature and spectral power changes, we separately analyzed the theta band. The correlation analysis revealed an 11 s lag (µ = 10.98 s, σ = 2.85 s) between the two signals (Fig. 3I). A similar delay was observed between the temperature and gamma power (Fig. S3B).

### Complex temperature dynamics during cataplexy

Our results show that temperature recordings can offer new insights into physiological sleep-wake states and brain activity. We next explored the temperature dynamics in the cortex during a non-physiological behavioral state, namely cataplexy. Instances of cataplexy, as observed in mice lacking the *hypocretin* gene (*Hcrt*^*−/−*^ mice), are ∼1 min long events following a sudden loss of muscle tone while awake^35^. Brain activity during cataplexy progresses through a sequence of distinct patterns starting with a brief phase (4–8 s) during which the EEG resembles that of the waking immediately preceding cataplexy onset, followed by a period (∼40 s) typified by high-amplitude and irregular theta activity of prefrontal cortical origin (referred to cataplexy associated state or CAS), and finally a phase of REM-sleep-like activity, especially during longer bouts of cataplexy, before resuming normal active wake behavior^21^.

**Fig. 4:**
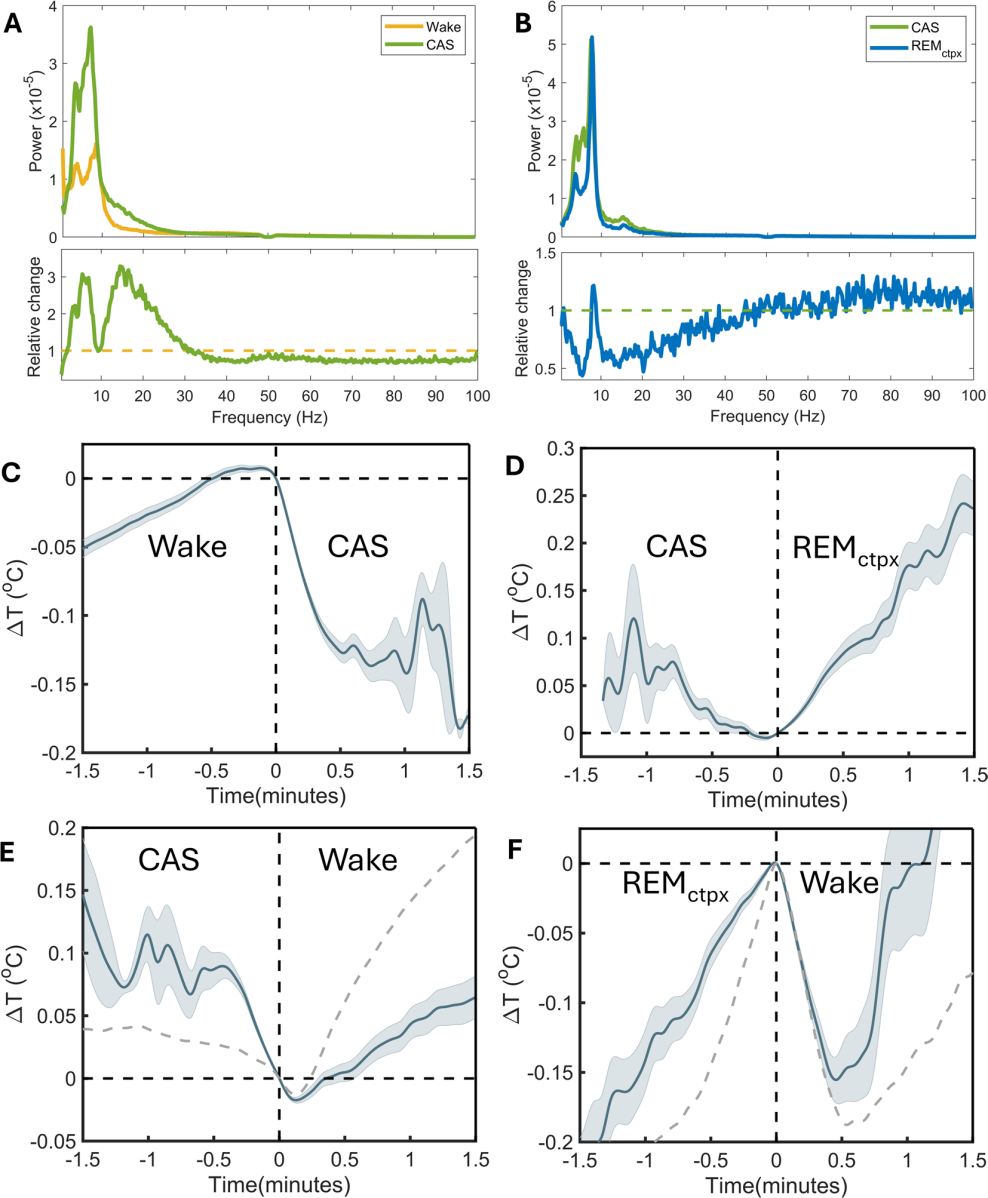
Temperature dynamics during cataplexy. (**A**) The power spectra averaged over 3 epochs (12 s) before and after the transition from wakefulness to CAS (upper panel). The bottom panel shows a ratio of CAS spectral power to wakefulness spectral power. Due to the increased theta power during wakefulness, the 8–10 Hz frequency band does not change during the transition. (**B**) The power spectra averaged over 3 epochs (12 s) before and after transitioning from CAS to the REM-sleep-like state during cataplexy. The bottom panel shows a ratio of REM-sleep-like state spectral power to CAS spectral power. The transition shows a decrease in all frequency ranges except for the theta and gamma bands. (**C**) Transitions from wakefulness to CAS are followed by a drop in cortical temperature. (**D**) Temperature increases at transitions from CAS to the REM-sleep-like state. (**E**) Temperature changes during the transition from CAS to wakefulness. Similar to transitions from NREM sleep (shown with a grey dash line), temperature starts increasing immediately after the state switch, but with a smaller magnitude, likely due to a higher starting temperature. (**F**) The temperature changes after the end of REM-sleep-like state. Following the transition to wakefulness, the temperature first decreases and then increases, similar to the transition from regular REM sleep to wakefulness (shown with a grey dash line).

Video-assessed instances of cataplexy were determined in 4 *Hcrt*^*−/−*^ mice equipped with EEG/ EMG electrodes and an epidurally placed thermistor. Each mouse was recorded for 48 h and a total of 249 cataplexy events were recorded, all occurring in the 12 h dark periods. In this dataset, we could verify the average sequence of EEG states after the onset of video-defined cataplexy: a wake-like state, CAS, and finally a REM-sleep-like state. The onset of cataplexy typically occurs during active waking behavior associated with high theta power^21^. Accordingly, our algorithm successfully identified 80% of the wakefulness before cataplexy as TDW. Although theta power was already high before cataplexy onset, the comparison between power spectra before and after the transition from waking (including TDW) to CAS, showed a further increase in theta power accompanied by a 1.5 Hz shift to a slower frequency (Fig. 4A). Additionally, we observed increases in sigma (11–15 Hz) and in the high delta (3–4 Hz) power (Fig. 4A). After longer CAS episodes (average duration of 42 s, Figure S4) animals typically exhibit a REM-sleep-like state during cataplexy. After the switch to the REM-sleep-like state, activity in the theta band (7.5–9 Hz) remains the same, while in all other frequency bands activity decreased (Fig. 4B) compared to that in CAS, resulting in an EEG signal closely resembling regular REM sleep.

We then wondered whether CAS, with its high theta content (Fig. 4A), is accompanied by a temperature increase similar to that of the theta-dominated states TDW and REM sleep. Contrary to expectation, we observed a sharp decay in cortical temperature after switching to CAS from wakefulness (Fig. 4C), similar to that observed during wake-to-NREM-sleep transitions (Fig. S1D). Cortical temperature again increased after transitions from CAS to REM-sleep-like state (Fig. 4D) as after physiological NREM-to-REM sleep transitions (Fig. S1B) although the rate of increase was smaller.

The moment *Hcrt*^*−/−*^ animals resume active waking behavior marks the end of cataplexy. Mostly, these transitions happen directly from CAS (~ 46% of cases) or from a REM-sleep-like state (~ 32% of cases) but were also observed from wakefulness (~ 17% of cases) and NREM sleep (~ 5% of cases). Note that cataplexy and sleep-wake state were determined independently, i.e., by video observation and annotation of EEG/EMG signals, respectively. Switching from CAS to wakefulness was followed by an increase in cortical temperature (Fig. 4E). Transitions from the REM-sleep-like state to wakefulness were followed by an initial steep decrease in brain temperature, followed by an increase (Fig. 4F), similar to the temperature dynamics observed during transitions from regular REM sleep to wakefulness (Fig. 4F - gray dashed line and Fig. S1).

## Discussion

The present work confirms the tight relationship between cortical temperature dynamics and sleep-wake states. We further illustrated this with a simple algorithm that predicted sleep-wake states based on the characteristic temperature changes associated with sleep-wake states. In addition, we show that brain temperature tracks EEG activities within sleep-wake states known to be associated with increased neuronal activity. Periods of increased sleep spindle prevalence during NREM sleep and variations in theta and gamma activity in REM sleep and TDW all were followed by increases in temperature. These increases occurred with a 10–14 s delay, independent of the type of EEG activity. Interestingly, similar delays were observed after stress and emotional stimuli^12^. Our results suggest that temperature changes directly report on increases in neuronal activity in the cortex either related to brain state or specific EEG activities typical of these brain states.

The observed response of cortical temperature to changes in neuronal activity may seem slow and question a direct link between the two. This delay could reflect the temperature propagation from deeper brain regions and thus the primary source of neuronal activity might not be cortical. Heat propagation distance can be estimated using a simplified equation for the thermal diffusion length $\:\mu\:\left(t\right)=\sqrt{4Dt}$, where *D* is a thermal diffusivity^36^. The thermal diffusivity for brain was estimated in calf and at a temperature of 41 °C is equal to 0.147 mm^2^/s^37^. Considering the 10–14 s delay we observed, the above equation gives a 2.4–2.9 mm distance from the point of measurement on the cortical surface which encompasses the subcortical regions involved in theta and spindle generation. However, it was shown that neurons reabsorb most of the generated heat thus precluding its dissipation over longer distances^38^. It seems therefore more likely that the temperature increases follow neuronal activity occurring in close proximity to the thermistor. Another factor that could have contributed to the 10–14 s delay concerns the fast increases in cerebral blood flow (CBF) resulting from the tight coupling between neuronal activity and local vasodilation^39,40^. Bergel et al.^28^ demonstrated increases in blood flow during REM sleep following surges in theta and gamma power with a delay of only 1.5–2.0 s. Additionally, sleep-wake related changes in CBF have been reported^41,42^. Typically, blood and body temperature are cooler than that of the brain, and CBF acts as a heat sink^2^. Therefore, vasodilation leads to a fast, local influx of cooler blood, which might contribute to the delay between neuronal activity, as quantified with the EEG, and the tissue heating we observed.

The relation of brain temperature to neuronal activity gave new insights into cataplexy. As opposed to the two other theta-dominated brain states, REM sleep and TDW, instead of an increase in temperature, transitions into CAS were associated with a decrease in temperature similar to that observed after wake-to-NREM-sleep transitions. It was previously shown that the theta activity during cataplexy originates from the prefrontal cortex^21^ and did not concern the hippocampus, the structure that strongly contributes to the theta activity captured by the EEG during REM sleep and TDW^32,33^. Above we argued that the measured temperature changes inform on local changes in brain activity. The temperature decreases we observed after CAS onset could therefore relate to a shift of the main source of neuronal activity away from the hippocampus (which is in close proximity to the position of the thermistor) during the TDW preceding CAS, to prefrontal areas during CAS. Alternatively, while sharing the frequency range with that of hippocampal theta, CAS-associated prefrontal theta activity could be associated with reduced neuronal activity and the large amplitude, irregular theta waves intermixed with slow waves, might be accompanied by a neuronal firing pattern more akin that of NREM sleep when alternating up and down-states result in a net reduction of neuronal firing and CBF^43,44^. The EEG spectral changes seem to support this; although CAS is not associated with increases in low delta power (1–2.5 Hz), EEG activity in other frequency bands, i.e., high delta (3–4 Hz) and sigma (11–15 Hz), increased towards the higher levels typical of NREM sleep. Moreover, the frequency of CAS’ theta is 1.5 Hz slower, less regular in appearance than that during REM sleep and TDW as it is interspersed with delta waves^21^.

The REM-sleep-like state that typically ends cataplexy resembles regular REM sleep. Besides its indistinguishable spectral EEG profile also the temperature dynamics are typical of regular REM sleep. Therefore, although cataplexy is a behaviorally defined state, it could be argued that cataplexy neurophysiologically ends with the end of CAS, after which the animal either resumes wakefulness or transitions into REM sleep. That CAS resembles NREM sleep and the REM-sleep-like state REM sleep is further supported by the, on average, 42s duration of CAS, which is in line with the finding that the probability of an NREM-REM sleep transition to occur is modulated by the ∼50s infra-slow oscillation originating from the locus coeruleus (LC)^45^. While LC activity is greatly reduced during cataplexy, especially at its onset^46^, rebound from the high LC activity prior to cataplexy is dynamically compatible with the infra-slow timing of the alterations during NREM sleep between sudden surges in noradrenaline, reaching awake levels, and periods of LC silence^45^. The dynamic relationship between LC activity with the initiation of REM sleep or the arousal that ends cataplexy, in addition to the resemblance of the temperature dynamics and some of the EEG features between CAS and NREM sleep, warrant further investigation.

Our analyses show that brain temperature is an information-rich signal. Not only does it mark transitions between different sleep-wake states, it also reports on the oscillatory EEG events known to be associated with increased neuronal activity. While recognizing that we did not directly measure neuronal activity in the brain circuits implicated in the generation of these EEG events, brain temperature could be used as an accessible proxy of this activity, offering new insights into non-physiological brain states, such as cataplexy.

## Materials and methods

### Data acquisition

Some of the data that contributed to this work were previously published^47^ and we refer to that publication for details on the methods. Here we briefly discuss data acquisition, surgical procedures, and experimental design. Data from 15 male C57BL6/J mice and 4 Orexin/Hypocretin knock-out (*Hcrt*^*−/−*^) mice were included. *Hcrt*^*−/−*^ mice, homozygous for the *Hcrt*^*tm1Ywa*^-allele^35^ were obtained by crossing mice heterozygous for the mutation. The mutation was brought onto a C57BL6/J background. All mice were housed individually under a 12:12 h light-dark cycle, with ZT0 and ZT12 corresponding to light and dark onset, respectively. Ambient temperature was maintained at 23°C and food and water were provided *ad libitum*. The surgery took place under deep xylazine (10 mg/kg) and ketamine (100 mg/kg) anesthesia. Analgesia was administered the evening before surgery and for three days afterwards (Dafalgan in drinking water; 200–300 mg/kg). Electroencephalograms (EEGs), recorded from a frontal-parietal derivation, and electromyograms (EMGs), recorded from the neck muscles, were collected using Somnologica 3 (Embla) software. A 0.0625 Hz high-pass filter and a 50-Hz notch filter were applied to remove the DC signal and interference from surrounding electrical equipment. Both signals were used to label the sleep-wake states ’wakefulness’, ’NREM sleep’, and ’REM sleep’, at a 4 s resolution^30^. Sleep-wake states marked as having EEG artifacts were excluded from the EEG spectral analyses but included in the temperature and time-in-sleep-wake-state analyses. Brain temperature was measured by a thermistor (P25BA102J, Amphenol Advanced Sensors) placed on top of the right visual cortex corresponding to the mid-point of the frontal-parietal EEG electrode pair on the left hemisphere and was sampled at 10 Hz. Thermistors were supplied with a constant measuring current ($\:{I}_{const}=\:100\:microA$), and voltage ($\:V$) was measured to calculate the resistance ($\:{R}_{t}\:=\:V/{I}_{const}$). Temperature was then calculated from the resistance using the calibration constants provided by the manufacturer^47^. A 48 h recording (starting at light onset) during which animals were left undisturbed was used in this work. All animal procedures outlined were carried out using the guidelines of Swiss federal law and were approved by the Ethical Committee of the State of Vaud Veterinary Office. The methods are reported in accordance with ARRIVE guidelines.

### Prediction of sleep-wake states from temperature

We developed an algorithm that predicts sleep-wake states based on changes in brain temperature. The process begins by identifying periods of wakefulness. We first detect continuous temperature drops lasting more than 5 s. The start of these temperature declines is labeled as the end of a wakefulness episode. To determine the start of the episode, we search for a temperature increase that brings the temperature to the level observed at the end of the wake period. The point at which this increase begins is marked as the start of the wake episode.

To detect REM sleep episodes, we first focus on identifying their onset. A sliding window of 2 s is moved across the recording in steps of 0.1 s. For each window, a sigmoid function is fitted to the data. The start of any window where the fit produces an R^2^ value greater than 0.97 is marked as a potential REM sleep episode start. If two potential start points are within 3 s of each other, the later one is discarded. The end of the REM sleep episode is identified by finding the first continuous temperature drop longer than 0.4 s after the start point. Because every REM sleep episode is followed by at least one epoch of wakefulness, the 4 s after the REM sleep end is labeled as wake. Lastly, data not labeled as wakefulness or REM sleep are classified as NREM sleep.

### TDW detection

We developed an algorithm that analyses EEG signals using the continuous wavelet transform (CWT) and identifies epochs of waking that meet the TDW criteria (Fig. S2), defined earlier^48^. First, CWT is calculated using the MATLAB cwt function (with Shannon complex wavelet ‘shan0.5-2’, for frequencies between 0 and 100 Hz with a step of 0.25 Hz) (Fig. S2A). We then determine the highest peak between 3.5 and 15 Hz for every CWT spectrum. If the peak is located between 6.5 and 12 Hz, relative θ-power is obtained by calculating the ratio between the power in a dynamic theta band (observed θ peak ± 1 Hz) and the total power between 3.5 and 45 Hz (Fig. S2B). The threshold for detecting high θ-power is estimated from the analysis of REM sleep, a theta-rich state. We constructed an inverse cumulative distribution function (1-CDF) of the relative θ-power extracted from all REM sleep episodes (Fig. S2C). The threshold is then determined as the θ-power at the 95th percentile of the obtained function. The threshold was calculated separately for the 12 h light and dark periods as they differed and improved the agreement with the results of an previous version^48^. Waking time points with θ-power higher than threshold were considered as ‘high θ’. The time points were then binned into 4s epochs and labeled as TDW when containing > 25% of high θ time points. The epochs labeled as TDW are then further filtered according to 3 additional criteria: (1) the preceding epoch was scored either as regular waking or TDW; (2) the epoch immediately following was not scored as NREM sleep; (3) 4 s TDW epochs not followed or preceded by TDW were excluded.

The previous method was designed for C57BL/6J mice^48^ and we noticed that its performance varied with genetic background. We therefore tested the applicability of our new method in 3 inbred (C57BL/6J, DBA/2J, and C3H/HeJ) and 2 outbred (Crl: SKH1-*Hr*^*hr*^ and Crl: CFW (SW)) lines of mice for which we had previously recorded EEG/EMG signals and for which 4 s epochs of TDW were manually annotated specifically for the purpose of testing the new algorithm. Algorithmically and manually annotated TDW epochs were compared and for all strains a good agreement was obtained (Fig. S2D).

### Identification of cataplexy and cataplexy-associated state (CAS)

Cataplexy and CAS were identified as previously published^21^. Briefly, cataplexy was detected by video examination alone as abrupt body collapse during a high-motivation activity. Scoring was performed independent of any information concerning EEG activity. Criteria were as follows: *“(i) starts with an abrupt and global postural collapse; (ii) directly follows ≥ 40 s of intense*,* goal-oriented behaviour*,* showing one or several of the following activities: excited ambulation/exploration*,* rearing/jumping*,* burrowing*,* nest building*,* vigorous grooming or drinking; (iii) consists in ≥ 10 s of continuous immobility; and (iv) ends with sudden resumption of visible tone and purposeful behaviour*”^21^. All 4 *Hcrt*^*-/-*^ mice used in this work showed episodes of cataplexy.

CAS was detected by visual inspection of the EEG and is characterized by irregular, mixed delta/theta EEG oscillations, with a theta band (~ 7 Hz) dominating (see Fig. 4F)^21^. CAS differs from NREM sleep by theta dominance and differs from REM sleep by a highly irregular and large EEG amplitude. CAS is typically observed during cataplexy in *Hcrt*^*-/-*^ mice and was not observed in wild type animals.

### Spectral analysis

We used 3 methods to perform spectral analysis. To detect significant rhythms in temperature and sigma power we used Lomb-Scargle periodogram (Fig. 2A) due to built-in significance metric. To calculate the spectra, we used MATLAB *plomb* function with oversampling factor of 4. The significance cutoff was calculated at p-value of 0.05. Peaks higher than the significant level were selected for further analysis. Before the spectral analysis, temperature recordings from NREM sleep were detrended by fitting and subtracting the second order polynomial and we subtracted the mean from EEG sigma power.

For cross-correlation analysis and TDW detection, we used continuous wavelet transform (CWT) method (Figs. 2, 3 and 4, S2, S3, S4). CWT was calculated using cwt MATLAB function with Shannon complex wavelet ‘shan0.5-2’, for frequencies between 0 and 100 Hz with the step of 0.25 Hz. EEG signals were not additionally modified for the analysis.

For spectral changes in the EEG during state transitions, we used the Discrete Fourier transform method (Somnologica package in MATLAB). Spectra were calculated for each 4 s epoch for frequencies between 0 and 100 Hz (Nyquist frequency) with 0.25 Hz resolution. The notch filter was used to remove frequencies between 49 and 51 Hz due to electrical equipment noise.

### Transition analysis

We analyzed temperature and spectral changes during state transitions. First, we selected transitions where animals spent in one state at least 3 epochs (12 s) before the transition and 8 epochs (32 s) after the transition. The selected episodes were aligned by the transition point. Only the last episode before the transition and the first episode after the transition were used in average temperature change calculation. For the calculation, for each episode, the temperature at transition point was subtracted from the temperature recording, thus producing relative changes $\:{\Delta\:}T$. The obtained $\:{\Delta\:}T\:$from multiple animals than averaged together (first within and then among individuals) to obtain the average temperature changes during state transitions. For spectral analysis, we calculate average spectral power change. For each frequency, we calculate the average power over 10 epochs (40 s) before the transition. The normalized power spectra than averaged over multiple animals and combined on a heatmap plot.

### Cross-correlations

The cross-correlation was used to detect the time delay between brain temperature and EEG spectral power. Changes in spectral power were calculated using continuous wavelet transform (CWT). The CWT was calculated the same way as described above. Depending on the analysis, the CWT power spectra were integrated over the frequency range of interest. The obtained power was then binned to a 10 Hz sampling rate to match the temperature recordings. Binned power was smoothed using a 20s moving window to reduce random variations. Temperature recordings were detrended to remove slower background fluctuations. For NREM sleep and wakefulness analysis, temperature was detrended by fitting and subtracting the second-order polynomial. For REM sleep analysis, sigmoid function was fitted and subtracted from the recording. Cross-correlations were calculated using the MATLAB *crosscorr* function (with the number of lags 1000, equal to 100 s).

## Supplementary Information

Below is the link to the electronic supplementary material.

Supplementary Material 1

## Data Availability

The datasets analyzed in the current study are available\u0000in the FigShare repository, complete with data descriptors and necessary code at [https://doi.org/10.6084/m9.figshare.29909831](https:/doi.org/10.6084/m9.figshare.29909831) .
