# Supplementary material for: Brain temperature as proxy for brain state and oscillatory activity in the mouse
Source: Sci Rep. 2025 Oct 24;15:37301. doi: 10.1038/s41598-025-21175-3 (PMC12552608; doi:10.1038/s41598-025-21175-3)
Supplement: Supplementary file 1 — Supplementary Material 1 [file 41598_2025_21175_MOESM1_ESM.docx]

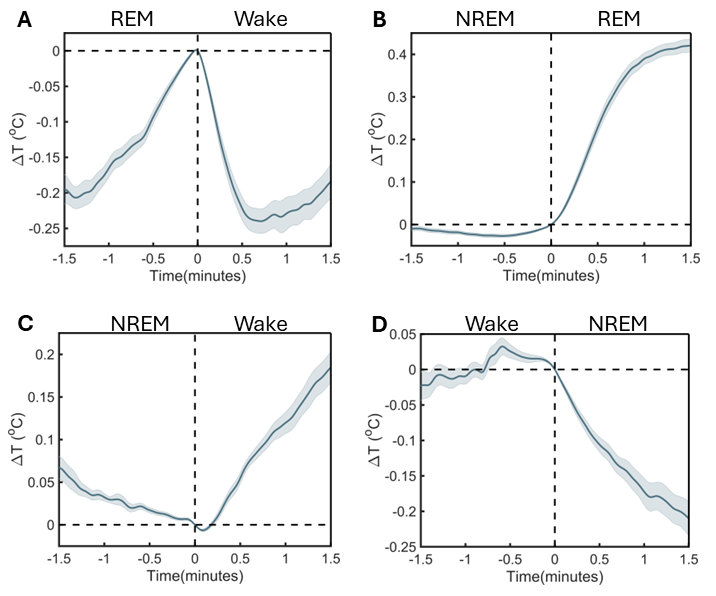


**Figure S1. Average temperature changes during transitions between sleep-wake states were calculated from 15 C57BL/6J mice**. The temperature at the transition point was subtracted before averaging. The transition point is indicated with the vertical dash line. The shaded area shows delimit +/- 1 SEM. The plots show transitions from REM sleep to Wake (A), NREM sleep to REM sleep (B), NREM sleep to Wake (C), and Wake to REM sleep (D).


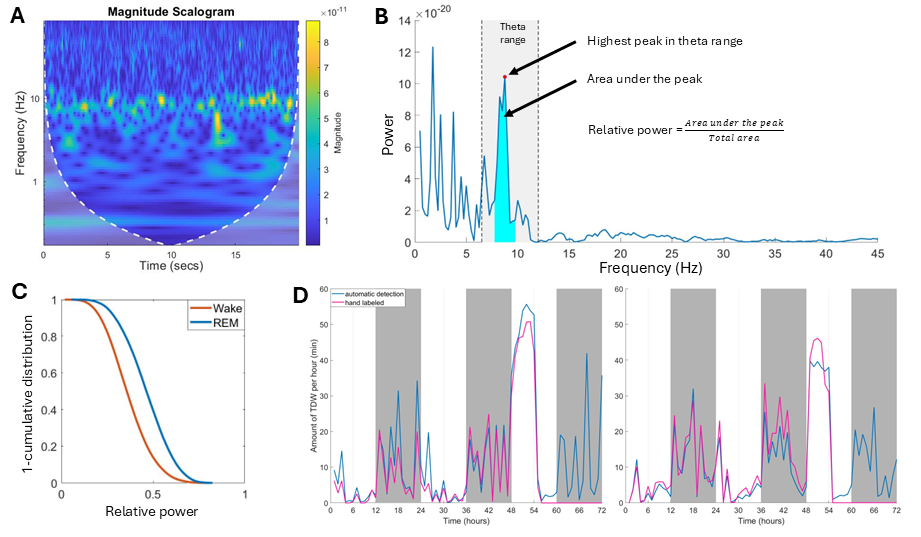


**Figure S2. TDW detection algorithm that utilizes continuous wavelet transform (CWT).** **(A)** First, CWT is calculated for the EEG signal with a Shannon complex wavelet, for frequencies between 0-100Hz with a 0.25Hz step. **(B)** For each CWT spectrum, we detect the highest peak between 3.5 and 15 Hz. If the peak is located between 6.5 and 12 Hz, the relative θ-power is obtained by calculating the ratio of the power in the θ peak ± 1Hz to the total power between 3.5 and 45 Hz (i.e., ‘Area under the peak’ and ‘Total area’ in the equation, respectively). **(C)** The threshold for detecting high θ-power is derived from the inverse cumulative distribution function (1-CDF) of the relative θ-power extracted from REM episodes. The threshold was set at the 95th percentile of the obtained function. Waking EEG with θ-power above threshold are considered as high θ time points. The time points are then binned into 4-second epochs by labeling the epochs with more than 25% of high θ time points as TDW. **(D)** To assess the accuracy of the algorithm, we compared its performance to manually annotated TDW (see Methods). The method accurately predicts TDW in different genotypes including C57BL/6J mice (left) and DBA/2J mice (right).


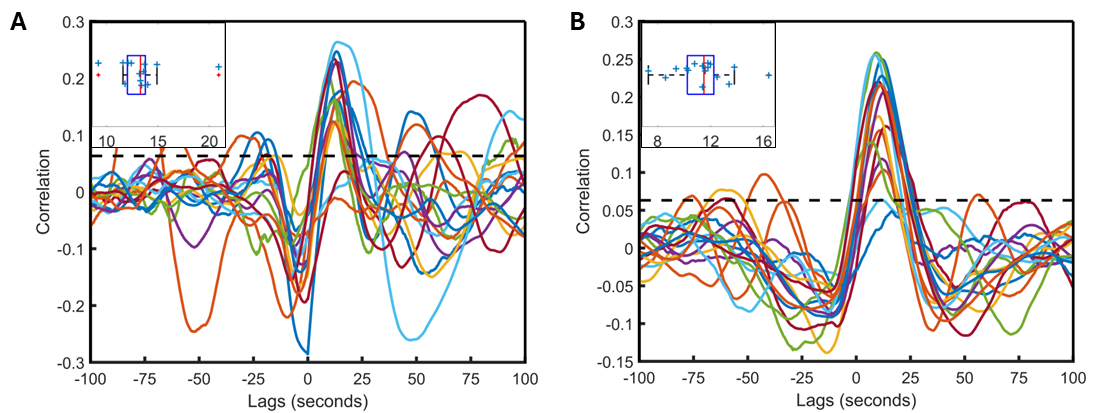
**Figure S3. Cross-correlation analysis with power changes in the gamma frequency band (55-80Hz).** **(A)** Cross-correlation between temperature and gamma power during REM episodes. The analysis shows an average delay of ~13.4 seconds (µ = 13.39s, σ = 2.66s). The observed delay did not differ from the delay calculated for theta frequency band (two sample T-test, p=0.26; see Figure 3E). **(B)** Cross-correlation between temperature and gamma power during wakefulness. We found a delay of 11.30s (µ=11.29s, σ = 2.14s) similar to the delay calculated for theta frequency band (two sample T-test, p=0.75; see Figure 3I). Peaks larger than $2/\surd N$ (dashed lines, where N is number of data points, N=1000 for the shortest considered episode), show a significant correlation.


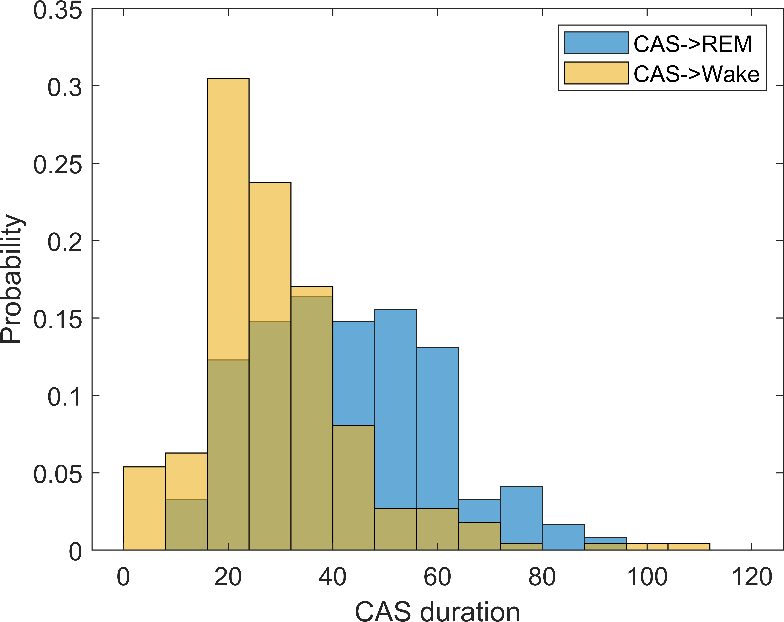


**Figure S4. Distributions of CAS episode durations that are followed by REM-sleep-like state and wakefulness.** REM-like state typically occurs after longer CAS episodes, with an average duration of 42 seconds.
